# Carcass Characteristics and Meat Quality of Wild-Living Mallard (*Anas platyrhynchos* L.) Originating from Croatia

**DOI:** 10.3390/foods13101519

**Published:** 2024-05-13

**Authors:** Nikolina Kelava Ugarković, Dalibor Bedeković, Kristina Greiner, Nera Fabijanić, Zvonimir Prpić, Miljenko Konjačić

**Affiliations:** 1 Division of Animal Science, Department of Animal Science and Technology, Faculty of Agriculture, University of Zagreb, Svetošimunska cesta 25, 10000 Zagreb, Croatia; 2 Division of Animal Science, Department of Animal Nutrition, Faculty of Agriculture, University of Zagreb, Svetošimunska cesta 25, 10000 Zagreb, Croatia; 3 Croatian Federation of Pig Breeders Associations, Ilica 101, 10000 Zagreb, Croatia; 4 Croatian Hunting Federation, Vladimira Nazora 63, 10000 Zagreb, Croatia

**Keywords:** mallard, carcass parts, meat color, pH, water-holding capacity, Warner–Bratzler shear force, proximate chemical composition, fatty acids, nutritional lipid indices

## Abstract

The aim of this study was to determine the effects of sex and hunting location on carcass characteristics and meat quality of wild-living mallard (*Anas platyrhynchos*) from Croatia. Twenty-eight mallards (14 ♂; 14 ♀) were hunted at two hunting locations (HL I = 8 ♂, 8 ♀; HL II = 6 ♂, 6 ♀) in the Croatian lowlands. The carcasses were eviscerated, dressed, and dissected, and the individual internal organs and carcass parts were weighed. The breast muscle (*m. pectoralis major*) was sampled and used to determine color, pH, drip loss, cooking loss, shear force, and proximate chemical and fatty acid composition. Sex and HL had a significant effect on the majority of carcass characteristics analyzed, but they had no effect on the physical meat parameters. The protein and ash content of mallard meat was significantly higher in HL II (23.16% vs. 22.67%; 1.45% vs. 1.36%, respectively) and the moisture content in females (72.40% vs. 71.59%). HL had a significant effect on SFA (II 33.96% vs. I 29.91%), PUFA n-3 (II 3.55% vs. I 2.69%), PUFA/SFA and n-6/n-3 ratios, and all lipid indices. Females had a significantly higher C22:6n-3 content, a higher PI index and a lower n-6/n-3 ratio. The data presented in this study contribute to a better understanding of game-bird meat quality originating from different regions.

## 1. Introduction

Game birds include a large group of birds that are classified as land fowls and water birds, including species such as partridges, quails, pheasants, ducks, geese, pigeons and snipes [1]. Throughout history, game birds have been hunted for recreational purposes but have also served as a source of food. Due to the increasing interest in food originating from wild, non-farmed species and sustainable systems, game birds have received more attention as a potential source of meat in the last decade [1,2].

Wild duck or mallard is one of most numerous and widely distributed dabbling duck species in the world, whose global population is estimated at 20 million individuals [3]. The population size in the Europe is estimated to 5.7–9.2 million mature individuals, inhabiting the wetlands of Western France, Middle Europe, Northern Italy and South-Eastern European countries [4]. Mallard is an omnivorous and opportunistic species that beside dabbling in water tend to graze, and their diet includes seeds, vegetative parts of crops and aquatic plants, terrestrial and aquatic invertebrates (insects, molluscs, crustaceans, worms and occasionally amphibians and fish) [4]. It is one of the most harvested game species worldwide, with an annual harvest in the European Union of up to 4.5 million individuals [5,6].

The quality of meat from different game birds varies greatly and is influenced by various factors. The meat of some game birds is very similar to that of chicken and consists mainly of white meat, while others have a stronger “gamey” flavor and may contain more dark meat [1]. Mallard meat is considered a delicacy, but in many countries, it is only consumed by hunters and their families [7]. Therefore, it is important to examine and analyze the quality of mallard meat from different regions. In addition, sexual dimorphism makes it easy to distinguish males from females, and the effect of sex may be important for meat quality. To date, several studies have presented data on the meat quality of mallards [7,8,9,10,11] from different parts of the world.

In developed countries, consumers are nowadays highly interested in the origin of food, production systems and their impact on the environment as well as on the nutritional properties of food and its health benefits. Game meat, due its characteristics, can fulfil all these requirements expected by modern consumers. Regarding food origin and production systems, wild game meat is considered more organic and sustainable than meat of domestic species and contributes to utilizing local food resources [10,12]. The nutritional properties of game meat are favorable due to the high protein content (up to 25%), low fat content (less than 5%), higher share of preferable fatty acids such as monounsaturated (MUFAs) and polyunsaturated (PUFAs), and better fatty acid ratios (especially n-6/n-3 ratio) [13]. Namely, PUFAs are associated with health-promoting benefits, i.e., they decrease blood cholesterol and potentially reduce the risk of coronary-heart diseases. In general, game meat is rich in PUFAs like C18:2n-6, C18:3n-3, C20:5n-3 and C20:4n-6, but their shares can differ depending on species, age, habitat, diet and season [13]. Different shares of n-6 and n-3 PUFAs also result in the different ratios and lipid indices of meat. The meat quality of large game species has been analyzed more over the past decade, while meat of small game and game-bird species is still not investigated much. Thus, it seems important to analyze meat of different game species from different regions to determine possible variations in quality, i.e., differences in the nutritional and health-related properties of meat. In Croatia, the mallard is one of the 23 game-bird species, and harvesting is carried out in accordance with legal regulations [14,15]. The species is mainly found in the continental parts of the country, e.g., in natural lakes, freshwater fish farms, river backwaters, streams and drainage channels, river deltas and even ponds [16]. The quality of the carcass and meat has not yet been studied in detail.

The aim of this study was to determine the effect of sex and hunting location on the carcass characteristics, color, pH, water-holding capacity, tenderness, chemical composition, fatty acid profile and lipid indices of mallard meat from the Croatian mainland. The data presented in this study should provide a better insight into the quality and nutritional value of mallard meat from different geographical regions of the world.

## 2. Materials and Methods

### 2.1. Study Area

The mallards used in this study were shot during the 2022 hunting season by licensed hunters in accordance with legal regulations. In the Republic of Croatia, mallard hunting is prohibited from February 1 to August 31 [15]. A total of 28 wild, free-living mallards were hunted during November and December of 2022.

**Figure 1:**
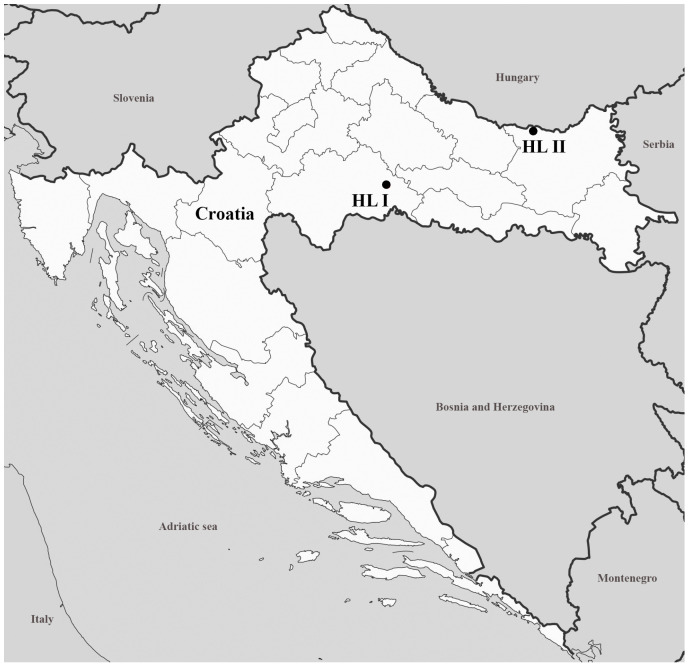
Map with marked hunting locations of wild mallards used in the study.

Sixteen (eight ♀ and eight ♂) individuals originated from Sisak-Moslavina County, Opeke II State Hunting Area (45.37300720043329, 16.819469719027822), marked as hunting location I (HL I) in the study (Figure 1). The most important big game species in the hunting ground are red deer (*Cervus elaphus*), roe deer (*Capreolus capreolus*) and wild boar (*Sus scrofa*). The Opeke II state hunting ground covers 8342 ha, borders the Lonjsko polje Nature Park and is located in the alluvial plain of the Sava River in the central basin of the Sava River. The area is surrounded by flooded forests of pedunculate oak (*Quercus robur*) and narrow-leaved ash (*Fraxinus angustifolia*), and corn, barley, triticale and wheat are grown on the adjacent agricultural land.

Twelve (six ♀ and six ♂) individuals originated from Osijek-Baranja County, Orahovica Farm, Donji Miholjac freshwater fish farm (45.75311436152088, 18.230133220618185), marked in the study as hunting location II (HL II) (Figure 1). The Donji Miholjac fish farm is part of a complete natural system of classic carp fish farms of Orahovica PP, which extends over 1017.9 ha, and the River Drava provides the unlimited water needed for production. The fish farm uses corn, wheat, barley (76% of the total) and pelleted feed (24% of the total) to feed the fish, which is distributed via automatic feeders at the edge of the ponds. In total, around 1770 tons of feed is used annually in the fish farms. The vegetation in fish farms consists of reeds, sedges, grass and white willow (*Salix alba*).

### 2.2. Sampling and Carcass Measurements

Within one hour post-mortem, each bird was transported to the laboratory in a CoolFreeze CDF 36 freezer (Dometic WAECO, Germany). In the laboratory, the mallards were individually weighed, skinned, dressed and dissected. Before skinning, the carcasses were decapitated at the atlanto-occipital joint, the wing tips were cut at the carpal joint (wrist), and the feet were cut at the ankle joint. Each part of the carcass was weighed using a JET 12002G/00 balance (Mettler Toledo, Greifensee, Switzerland). Skinning was performed by cutting the skin with feathers along the sternum and gradually removing it from the carcass. Each skin with feathers and tail was weighed individually. All internal organs were removed from the carcass as follows: Heart, liver, gizzard, and gastrointestinal tract. From the carcass, we dissected breast muscles, whole legs, neck (between last cervical vertebra and notarium), two-joint wings (drumette + mid-joint wing), which were weighed individually (JET 12002G/00, Mettler Toledo, Greifensee, Switzerland). Abdominal fat was considered to be all visible fat that could be easily separated from the abdominal cavity and organs. The bones remaining from the carcass after removal of the wings, whole legs, breast muscle and neck were weighed as trunk bones. The weight of the wing tips, two-jointed wings, feet, whole legs and breast muscle was presented as the sum of the left and right carcass parts. Both breast muscles were individually vacuum packed and stored at −20 °C until analyzed.

### 2.3. Determination of Physical Traits of Mallard Meat

The left breast muscle (*m. pectoralis major*) was used to determine the color, pH, drip loss of thawed samples, cooking loss and Warner–Bratzler shear force of mallard meat.

#### 2.3.1. Drip Loss of Thawed Samples

Frozen samples of left breast muscles were weighed (JET 12002G/00, Mettler Toledo, Greifensee, Switzerland) and placed on a polypropylene net in a plastic container in a refrigerator at 4 °C for 22 h. After this time, the samples were removed from the container, placed on a paper towel, blotted dry with paper towels, and weighed again, and the thawing loss was determined as follows:

Drip loss of thawed samples (%) = ((weight of frozen samples − weight of thawed samples)/weight of frozen samples) * 100

Thawed left breast muscle samples were used to determine other physical meat parameters.

#### 2.3.2. Cooking Loss

The cooking loss of mallard meat was determined as described by Honikel [17]. Weighed samples (approx. 50 mm thick and 50 g) were placed in thin-walled polyethylene bags, immersed in the preheated (80 °C) Sub Aqua Pro water bath (Grant Instruments Ltd., Cambridge, UK) and cooked until 75 °C was reached in the center of the sample. A Fantast digital meat thermometer with a probe (Ikea, Sweden) was used to measure the temperature of each sample. Afterwards, the samples were immediately immersed in an ice water bath for 10 min and then placed in the refrigerator at +4 °C until they cooled down. The samples were then removed from the bags, blotted dry with paper towels and weighed. The cooking loss is expressed as a percentage of the original sample weight as follows:

Cooking loss (%) = ((weight of samples before cooking − weight of samples after cooking)/weight of samples before cooking) * 100

#### 2.3.3. Meat Color and pH

The pH value of the meat was determined with a portable pH meter (S2-Food Kit, Mettler Toledo, Greifensee, Switzerland) and is presented as the average of three consecutive measurements.

Meat color was measured using the Chromameter CR-410 (Konica Minolta, Tokyo, Japan) with a 50 mm measuring surface and D45 illumination. Measurements are presented in CIELAB color space, including L* (lightness = 0–100, a* (redness = +60 red to −60 green) and b* (yellowness = +60 yellow to −60 blue). The meat color was measured in air 15 min after the meat was sliced.

#### 2.3.4. Warner–Bratzler Shear Force

The tenderness of the meat was determined according to Wheeler’s protocol [18] using the texture analyzer TA-XT Plus (Stable Micro Systems Ltd., Godalming, UK). The samples used to determine cooking loss were used in this analysis. The shear force was determined based on an average of at least three shears of the meat cores.

### 2.4. Proximate Chemical and Fatty Acid Composition

The right breast muscle was used to determine proximate chemical composition and fatty acid composition, as previously described by Kelava Ugarković et al. [19], according to ISO standards for moisture [20], fat [21], protein [22], ash [23] and fatty acids [24].

The sums of fatty acids were calculated as follows:

SFA = ∑(C14:0 + C15:0 + C16:0 + C17:0 + C18:0 + C20:0 + C22:0)

MUFA = ∑(C14:1 + C16:1 + C20:1 + C18:1n-9)

PUFA = ∑(C18:3n-3 + C20:3n-3 + C20:5n-3 + C22:6n-3 + C18:2n-6 + C20:3n-6 + C20:4n-6)

UFA = MUFA + PUFA

PUFAn-6 = ∑(C18:2n-6 + C20:3n-6 + C20:4n-6)

PUFAn-3 = ∑(C18:3n-3 + C20:3n-3 + C20:5n-3 + C22:6n-3)

PUFA/SFA = ratio between PUFA and SFA

n-6/n-3 = ratio between PUFAn-6 and PUFAn-3

The lipid indices presented in this study are as follows: atherogenicity index (AI), thrombogenicity index (TI), hypocholesterolemic/hypercholesterolemic ratio (h/H), peroxidability index (PI), nutritional value index (NVI) and health-promoting index (HPI) [25,26,27,28,29].

AI = (c12:0 + 4 * C14:0 + C16:0)/[(∑MUFA + ∑(n-6) + ∑(n-3)]

TI = (C14:0 + C16:0 + C18:0)/[(0.5 * ∑MUFA + 0.5 * (n-6) + 3 * (n-3) + (n-3)/(n-6)]

PI = (0.025 * monoenic%) + (1 * dienic%) + (2 * trienic%) + (4 * tetraenic %) + (6 * pentaenic%) + (8 * hexaenoic%)

h/H = [(C18:1n9 + C18:2n6 + C18:3n3 + C20:4n6 + C20:5n3 + C22:5n3 + C22:6n3/(C14:0 + C18:0)]

NVI = (C18:0 + C18:1n-9)/C16:0

HPI = (∑UFA)/(C12:0 + (4 * C14:0) + C16:0)

### 2.5. Statistical Analysis

The Shapiro–Wilk test in the SAS software V9.4 (Cary, NC, USA) [30] was used to test the homogeneity of distribution and variance. One-way ANOVA was used to analyze data that showed a normal distribution, while the Kruskal–Wallis test was used for data that did not show a normal distribution. Significance was tested at *p* < 0.05.

## 3. Results

This study presents the results of the carcass characteristics, physical properties, proximate chemical composition, fatty acid profile and lipid indices of mallard meat in relation to sex and hunting location.

### 3.1. Mallard Carcass Characteristics

The results of the mallard carcass characteristics in relation to sex and hunting location are presented in Table 1. Male mallards had a higher (*p* = 0.0001) carcass cold weight, head and neck weight, whole legs (*p* = 0.0002), breast muscle (*p* = 0.0003) and trunk bones (*p* = 0.0004) than female mallards. Body weight and two-joint wing weight were also higher in males than in females (*p* = 0.001), as were skin (*p* = 0.019) and feet weight (*p* = 0.012). The dressing percentage was very similar in males and females (*p* = 0.376) and was 51%. Mallards from HL I showed higher values for body weight (*p* = 0.002), neck (*p* = 0.001), skin (*p* = 0.005), wing tips (*p* = 0.029), two-joint wings (*p* = 0.032), feet (*p* = 0.024), gastrointestinal tract (*p* = 0.051) and abdominal fat (*p* = 0.039). Mallards from HL II had a higher (*p* = 0.002) dressing percentage than those from HL I. The interactions between sex and hunting location were significant for most of the analyzed mallard carcass characteristics (in Table 1 is presented level of significance SxHL). Here we describe only some of the interactions. The body weight of male mallards (1406.25 g) from HL II was higher than the body weight of female mallards from HL I (125.84 g; *p* < 0.05) and HL II (1069.00 g; *p* < 0.0001). In addition, the males from HL I had a higher cold carcass weight than the females from HL I (708.60 g vs. 609.77 g; *p* < 0.005) and HL II (708.60 g vs. 574.22 g; *p* < 0.0004). The same interaction was found for breast muscle weight (♂ HL I 263.91 vs. ♀ HL I 229.81 g, *p* < 0.05; (♂ HL I 263.91 vs. ♀ HL I 222.48 g, *p* < 0.01).

### 3.2. Physical Characteristics of Mallard Meat

Table 2 presents the results of the physical characteristics of the mallard meat in relation to sex and hunting location. Sex and hunting location showed no influence (*p* > 0.05) on the analyzed physical parameters. Mallard meat is found to be dark red (average L* 29.21 and average a* 13.81) and tough (average shear force 53.49 N/m^2^), with an average cooking loss of 11.17% and an average drip loss of the thawed samples of 7.16%.

### 3.3. Proximate Chemical Composition of Mallard Meat

The proximate chemical composition of the mallard meat is presented in Table 3. The meat of female mallards had a higher moisture content (*p* = 0.049) than that of male mallards. In relation to hunting location, the meat of mallards from HL II had a higher protein (*p* = 0.028) and ash (*p* = 0.006) content than the meat of mallards from HL I. The interactions between the analyzed effects were significant (*p* < 0.05) for protein, fat and ash content. Thus, meat from males from HL II had a higher protein content than meat from males from HL I (23.67 vs. 22.73%) and from females from HL I (23.67 vs. 22.61%). Males from HL I had a higher fat content than males from HL II (4.59 vs. 3.50%) and females from HL II (4.59 vs. 3.68%).

### 3.4. Fatty Acid Composition and Lipid Indices of the Mallard Meat

The results of the fatty acid composition, the qualitative indices of the fatty acid composition and nutritional lipid indices of the mallard meat are presented in Table 4. The predominant fatty acid in mallard meat was C16:0 (about 19%), followed by C18:0 (about 11%). The most abundant MUFA was C18:1n-9, which accounted for about 34% of the total fatty acids. Among the PUFAs, C18:2n-6 was the most abundant, accounting for about 19% of the total fatty acids, followed by C20:4n-6 (about 9%). MUFAs were the most abundant fatty acids in mallard meat (37%), while PUFAs and SFAs had almost the same proportion (32% and 31%, respectively). Sex had a minor influence on the fatty acid composition and lipid indices. Thus, a higher (*p* = 0.027) C22:6n-3 content, a lower (*p* = 0.031) n-6/n-3 ratio and a higher (*p* = 0.018) PI index were found in the meat of female mallards. Differences between hunting locations were more evident, and mallard meat from HL II had higher C14:0 (*p* = 0.008), C16:0 (*p* = 0.032) and C18:0 (*p* = 0.001) content than that from HL I. Therefore, SFA content was higher in mallard meat from HL II (*p* = 0.0001). Regarding the interactions between the analyzed effects, the meat of male mallards from HL I had a lower (*p* = 0.05) C16:0 content than that of males from HL II, while females and males from HL I had a lower (*p* = 0.05) C18:0 and SFA content than males from HL II. The meat of mallards hunted in HL I had higher (*p* = 0.027) C18:2n-6 content but lower C20:5n-3 (*p* = 0.001) and C22:6n-3 (*p* = 0.006) content than the meat of mallards from HL II. This resulted in higher (*p* = 0.054) PUFAn-3 content and a lower (*p* = 0.037) n-6/n-3 ratio in the meat of mallards from HL II. Regarding the interactions between the analyzed effects, the meat of female and male mallards from HL I had lower (*p* = 0.05) C20:5n-3 and C22:6n-3 but higher (*p* = 0.05) UFA content than the meat of males from HL II. Lower AI (*p* = 0.003), TI (*p* = 0.021) and PI (*p* = 0.019) indices were found in mallard meat from HL I, while the h/H ratio was higher (*p* = 0.009) than in meat from HL II. The nutritional value index (NVI) and health promotion index (HPI) were very similar for meat from female and male mallards (*p* = 0.813; *p* = 0.928). A slightly higher NVI value was found for mallard meat from HL I compared to meat from HL II, while mallard meat from HL I had a higher HPI value (*p* = 0.006) than meat from HL II.

## 4. Discussion

This study presents various results regarding the carcass and meat quality of wild mallard—one of the most abundant wild bird species in the world. It was found that in the analyzed wild-mallard carcasses, the most valuable part—the breast muscle—accounted for about 38% of the carcass cold weight, and together with the whole legs, two-joint wings and neck (all with bones), accounted for up to 51% of the carcass cold weight. In the wild mallards from Croatia, the proportion of breast muscle in the total body weight is higher than in D11 Dworka and P9 Pekin ducks (19.3% vs. 16.8% and 15.2%, respectively) [31]. Szász et al. [32] reported a lower weight of breast muscle for female and male mallards (167 and 153 g vs. 226.22 and 261.23 g, respectively), while Janiszewski et al. [7] reported a higher weight of breast muscle than in the present study (313.7 and 278.8 g vs. 226.22 and 261.23 g, respectively).

Regarding the effect of sex, male mallards were found to have higher body weight, cold carcass weight and most other parts of the carcass. A similar effect of sex on mallard carcass characteristics was reported by Janiszewski et al. [7], but compared to the present study, the same authors reported lower body, liver and heart weight and higher weights of breast muscle, gizzard and legs. The differences observed may be attributed to possible age and nutritional differences in addition to sex-specific effects [33]. It is not possible to determine the age of wild-living mallards by visual inspection [12], so it only can be assumed. In the present study, differences in carcass characteristics were found depending on the hunting location, especially in some important carcass characteristics such as body weight, carcass cold weight, weight of breast muscle and whole leg (with bone), abdominal fat content and dressing percentage. Thus, it can be expected that mallards from different regions (within a country and between different countries) will have different proportions of valuable and less valuable carcass parts. In rural areas of developing countries, where people very often rely on wild game species as a source of animal protein, this may be an important factor in obtaining sufficient meat without negatively impacting wildlife populations (excessive hunting) [34].

Physical meat characteristics such as pH, color, drip loss, cooking loss and shear force are important indicators of meat quality. In the present study, very consistent values were found for these traits, with very similar values depending on sex and hunting location. Similar results on physical meat parameters in wild mallards were reported by other authors and in other duck species [7,12,31]. In the present study, similar pH values but lower values for color parameters (especially L* and a*), cooking losses and drip losses were found compared to Janiszewski et al. [7]. The reported values for pH, color parameters and shear force for D11 Dworka and P9 Pekin ducks were higher than in the present study [31]. Söderquist et al. [12] reported higher L* values and cooking losses for both farmed and wild mallards than in the present study, but the reported a* values were unusually lower than in the present study and for meat in general. Pekin and Muscovy ducks [35] were found to have higher L* and a* values, but lower b* values and almost twice lower shear force than in the present study. The observed differences in physical meat traits can be attributed to possible differences in age, intensity of muscle use and muscle fiber types [31] as well as to the methods used to determinate certain traits and whether used meat samples were fresh or frozen prior the analysis. Compared to pheasant meat [36,37], it seems that wild-mallard breast meat appears to have a similar pH but lower L* and b*, making it more “gamey”. The darker color can be attributed to a higher share of red fibers [7] and consequently higher myoglobin and haemoglobin content characteristic for wild game meats.

In the human diet, meat is primarily a source of protein but can also contribute significantly to total fat intake, depending on the species. The nutritional value of meat varies according to species, diet, age and muscle mass. In the present study, the protein content was similar to that found in mallard meat in previously published studies [7,8,10,12]. As in the previous studies, no significant influence of sex on the chemical composition was found. Thus, specimens of both sexes can be equally represented in the human diet and provide valuable protein content. The fat content showed major differences between the present and previously published studies. Namely, several earlier studies reported a lower fat content in mallard meat than in the present study. Janiszewski et al. [7] reported substantially lower fat content in mallard meat than in the present study (0.83% vs. 3.99%, respectively). Quaresma et al. [10] reported a lower fat content in the breast muscle of mallards reared under semi-extensive conditions (2.02% vs. 3.99%, respectively). Söderquist et al. [12] reported lower fat contents in farmed and wild mallards than in the present study (2.7–3.47% vs. 3.99%, respectively). The most similar fat content in the breast muscle of wild ducks to that of the present study was determined by Cobos et al. [8] (3.99% vs. 3.39%, respectively) and Krempa et al. [11] (3.99% vs. 3.69%, respectively). The observed differences in fat content can be attributed to age, diet, hunting season and habitat. It appears that the wild mallards used in the present study had a higher food supply, resulting in a higher fat content of the meat, which is more similar to that of domestic duck species [7,38,39]. The hunting location affected the protein and ash content in the present study, suggesting that the meat of mallard ducks from different locations may provide different amounts of essential nutrients.

As far as the nutritional value of meat is concerned, in addition to the fat content, the fatty acid composition is also of crucial importance. The modern consumer is aware that high levels of saturated fat in the diet can have a negative impact on health by causing cardiovascular disease and increased blood cholesterol levels. On the other hand, unsaturated fatty acids, especially n-3 PUFAs, have a positive effect, and the ratios between the main groups of fatty acids (SFAa, MUFAs, PUFAs) can be used to determine the nutritional value of meat. In addition to C16:0, C18:1n-9 and C18:2n-6, which account for more than 60% of the total fatty acids in mallard meat [7,8,9,10,40], mallard meat also contains valuable proportions of PUFAs, such as C20:4n-6 and C22:6n-3. Similar to the present study, Söderquist et al. [12] reported the highest levels of C18:1n-9 in both farmed and wild ducks, followed by C16:0, C18:2n-6 and C18:0, respectively. The levels of SFAa, MUFAs and PUFAs were also similar, but higher PUFAn-3 levels were found in the present study. Krempa et al. [11] reported a lower content of C18:1n-9 and C22:6n-3, a similar content of C16:0 and a higher content of C18:0 and C18:3n-3. The same authors reported a dominant proportion of SFAs in mallard meat, followed by PUFAs and MUFAs. Quaresma et al. [10] reported very similar results regarding fatty acid composition, with the exception of C18:2n-6 and PUFAs, which were lower than in the present study. Major differences were found with the results of Janiszewski et al. [7], who reported a higher C18:0, substantially lower C18:1n-9 and substantially higher C18:3n-3 and C20:4n-6 content. Thus, the same authors reported a substantially lower content of MUFAs and a higher content of PUFAs than in the present study. Similar results regarding the total sums of fatty acids were also reported by Cobos et al. [8]. Regarding the analyzed effects, sex had only a minor influence on the fatty acid composition, which was also reported by previous studies [7,10,12]. Sex-specific differences in fatty acid composition were reported by Bombik et al. [9]. The same authors reported a greater influence of hunting location on fatty acid composition, similar to the present study. Differences in the fatty acid composition of the meat of mallards from different regions, i.e., hunting locations, can be attributed to differences in diet. As omnivores, the diet of wild mallards includes numerous components, such as cereals, aquatic plants and animals, and grazing [8,40], and depending on the habitat and available food, the diet of mallards may differ from place to place, leading to differences in fatty acid composition. The main differences between the two hunting locations in the present study were habitat characteristics and available food. Namely, at HL II (carp fish farm), the mallard diet could have included maize and fish pelleted food that was distributed during fish feeding. Additionally, common carp are omnivorous and prefer animal food, such as water insects, larvae of insects, worms, molluscs, and zooplankton. Zooplankton is a dominant food source in fishponds, with a high stock density on fish farms [41], which was the case at HL II. Thus, all these food sources were available for mallards at HL II. At HL I, mallards stay on the backwater, wetland, and natural ponds and can forage agriculture crops and maize used for the supplementary feeding of large game species. Although maize was available at both hunting locations, it was found that different maize hybrids have different linoleic acid content and different effects on the meat fatty acid profile [42]. This could also be a reason for the different C18:2n-6 content in meat from mallards hunted at different locations.

Ratios of the fatty acids—PUFA/SFA and n-6/n-3—and nutritional lipid indices are used to determine the contribution to potential health risks and the nutritional value of dietary fat. In the present study, a favorable PUFA/SFA ratio was found as all analyzed sex and hunting groups had a ratio above the minimum recommended: 0.45. Recently, relevance of this ratio has been discussed, and it has been suggested that it is outdated as it does not include specific individual fatty acids and classes [43]. Namely, within the sum of PUFA and SFA, there are fatty acids with more and less favorable effects on human health, and when presented in sums, their effect may be less obvious. The dominant SFA in mallard meat, C16:0, is a cholesterol-increasing fatty acid when compared to C18:0, the second-most-abundant SFA. This is because C18:0 is thought to be converted to C18:1n-9 in the body, and this fatty acid appears to have a neutral effect on blood cholesterol levels [44]. Some previous studies [7,10] have found similar levels of PUFA/SFA in mallard meat, while Bombil et al. [9] reported lower levels. Due to the high levels of MUFA and PUFA, which account for two-thirds of the total fatty acids, the meat of wild mallards can be considered of high nutritional value compared to poultry genotypes [43], as wild duck meat has a lower content of SFA, a higher MUFA content and a similar PUFA content.

A favorable n-6/n-3 ratio should be below 4, and values 2-3x higher than recommended were found in the present study. A higher n-6/n-3 ratio in mallard meat was reported by Quaresma et al. [10], but lower values were reported by Janiszewski et al. [7] and Krempa et al. [11]. Compared to poultry meat, the values were in the range of the fast-growing chicken genotypes used in the study by Dal Bosco et al. [43]. The lowest n-6/n-3 ratio was found in mallard meat from HL II, due to the highest n-3 PUFA content being in this meat, showing that the nutritional value of mallard meat can be significantly influenced by habitat and diet. These fatty acids are derived from the diet but also from phospholipids, which are found in higher proportions in the meat of duck species [8].

The calculated lipid indices for mallard meat showed favorable values, as the AI and TI indices were below 1 and the h/H ratio was above 2.5 in all groups studied. Bombik et al. [9] reported similar values for the AI, TI and PI indices of mallard breast meat. Quaresma et al. [10] reported similar values for the AI index but higher TI and lower PI indices than in the present study. Higher PI values are the result of higher PUFA content, which indicates a higher susceptibility of mallard meat to peroxidation during its prolonged shelf life. Regarding the nutritional value of mallard meat, in the present study, both the nutritional value index and health-promoting index were higher than for poultry meat [43] and thus more favorable. The health-promoting index also showed differences in mallard meat from different hunting locations, indicating that greater variations in fatty acid ratios and indices are to be expected due to numerous possible influences such as diet, hunting location, season, age and migration.

## 5. Conclusions

Different quantities and qualities of meat can be expected depending on the sex and hunting location of wild mallards. Physical meat traits seem to be similar between sexes and hunting locations, and mallard meat can be characterized as dark meat with a higher shear force. The nutritional value of mallard meat is favorable due to the high protein content and preferable shares of fatty acids. Namely, MUFAs are the dominant group of fatty acids, while PUFAs make up at least one-third of the total fatty acid content. It seems that habitat and diet variations can result in significant differences in the content of essential n-6 and n-3 fatty acids like C18:2n-6, C20:5n-3, C20:6n-3, and C20:4n-6. Consequently, the nutritional lipid indices of mallard meat can differ between hunting locations and available food sources, but in general, they have favorable values in line with health authorities’ recommendations. A high share of unsaturated fatty acids can make mallard meat suspectable to lipid oxidation. Mallard meat has good nutritional potential either as a component of a varied diet or as a gourmet food.

## Figures and Tables

**Table 1:** Effect of sex, hunting location and their interaction on mallard carcass characteristics (LSMEAN ± SE).

Variable (g)	Sex (S)		Hunting Location (HL)		*p*		
	♀	♂	I	II	S	HL	SxHL
Body weight	1160.91 ± 29.73	1327.95 ± 34.98	1328.55 ± 27.64	1160.30 ± 37.61	0.001	0.002	*
Cold carcass	592.41 ± 13.43	696.17 ± 15.79	659.19 ± 12.48	629.39 ± 16.98	0.0001	0.171	**
Skin ^1^	267.31 ± 16.44	330.04 ± 19.34	339.00 ± 15.28	258.34 ± 20.79	0.019	0.005	*
Head	52.79 ± 1.04	64.04 ± 1.22	60.03 ± 0.96	56.81 ± 1.31	0.0001	0.060	**
Wing tips	73.98 ± 5.81	80.75 ± 6.83	87.97 ± 5.40	66.75 ± 7.35	0.451	0.029	ns
Feet	15.29 ± 0.41	17.02 ± 0.48	16.94 ± 0.38	15.37 ± 0.52	0.012	0.024	**
Gizzard	36.50 ± 1.78	40.62 ± 2.09	40.66 ± 1.65	36.47 ± 2.25	0.141	0.146	ns
Gastrointestinal tract	65.07 ± 2.73	66.65 ± 3.21	69.92 ± 2.54	61.80 ± 3.45	0.705	0.051	ns
Abdominal fat	25.53 ± 4.12	27.36 ± 4.64	32.19 ± 3.65	20.69 ± 5.22	0.765	0.039	ns
Trunk bones	187.06 ± 4.44	214.86 ± 5.23	207.22 ± 4.13	194.71 ± 5.62	0.0004	0.087	**
Neck	36.05 ± 1.07	44.30 ± 1.25	43.48 ± 0.98	36.88 ± 1.34	0.0001	0.001	***
Two-joint wing ^2^	49.75 ± 1.13	56.68 ± 1.34	55.30 ± 1.05	51.23 ± 1.44	0.001	0.032	**
Heart	15.01 ± 1.08	15.26 ± 1.27	13.54 ± 1.00	16.74 ± 1.36	0.874	0.072	ns
Liver	26.25 ± 2.14	26.09 ± 2.52	24.61 ± 1.98	27.74 ± 2.70	0.960	0.361	ns
Breast muscle	226.22 ± 5.45	261.23 ± 6.42	246.86 ± 5.06	240.59 ± 6.89	0.0003	0.472	**
Whole leg	81.05 ± 1.72	92.46 ± 2.02	88.84 ± 1.59	84.68 ± 2.18	0.0002	0.138	**
Dressing (%)	51.48 ± 0.85	52.65 ± 1.01	49.68 ± 0.79	54.44 ± 1.08	0.376	0.002	*

^1^ weight of skin with feathers and tail; ns = not significant; ^2^ weight of drumette and mid-join wing; * *p* < 0.05; ** *p* < 0.001; *** *p* < 0.00001; S = sex; L = location; SxHL = sex and hunting location interaction.

**Table 2:** Effect of sex, hunting location and their interaction on physical characteristics of mallard breast meat (LSMEAN ± SE).

Physical Parameters	Sex (S)		Hunting Location (HL)		*p*		
	♀	♂	I	II	S	HL	SxHL
pH	5.96 ± 0.06	5.87 ± 0.07	5.96 ± 0.05	5.88 ± 0.07	0.339	0.348	ns
L*	29.28 ± 0.43	28.92 ± 0.51	29.41 ± 0.40	28.79 ± 0.55	0.584	0.368	ns
a*	13.92 ± 0.42	13.83 ± 0.49	13.61 ± 0.39	14.14 ± 0.53	0.898	0.425	ns
b*	3.36 ± 0.31	3.61 ± 0.36	3.57 ± 0.29	3.40 ± 0.39	0.624	0.719	ns
Drip loss ^1^ (%)	7.09 ± 0.64	7.83 ± 0.75	6.57 ± 0.59	8.35 ± 0.81	0.459	0.091	ns
Cooking loss (%)	10.86 ± 0.69	11.30 ± 0.82	11.50 ± 0.65	10.66 ± 0.88	0.678	0.453	ns
Shear force (N/m^2^)	50.79 ± 2.83	56.20 ± 3.33	52.84 ± 2.63	54.15 ± 3.58	0.222	0.771	ns

^1^ of thawed samples; ns = not significant; SxHL = interaction between sex and hunting location; L*, a*, b* = lightness, redness and yellowness measured 15 min after exposing meat to air.

**Table 3:** Effect of sex, hunting location and their interaction on the proximate chemical composition of mallard breast meat (LSMEAN ± SE).

Chemical Composition (%)	Sex (S)		Hunting Location (HL)		*p*		
	♀	♂	I	II	S	HL	SxHL
Moisture	72.40 ± 0.27	71.56 ± 0.31	71.72 ± 0.24	72.24 ± 0.34	0.049	0.231	ns
Protein	22.74 ± 0.13	23.09 ± 0.16	22.67 ± 0.12	23.16 ± 0.17	0.090	0.028	*
Fat	3.72 ± 0.23	4.18 ± 0.27	4.20 ± 0.21	3.70 ± 0.29	0.204	0.177	ns
Ash	1.42 ± 0.02	1.38 ± 0.02	1.36 ± 0.02	1.45 ± 0.02	0.232	0.006	*

SxHL = sex and hunting location interaction; ns = not significant; * *p* < 0.05.

**Table 4:** Effect of sex, hunting location and their interaction on carcass characteristics of mallard breast meat (LSMEAN ± SE).

Fatty Acid Composition (% of Total Fatty Acids)	Sex (S)		Hunting Location (HL)		*p*		
	♀	♂	I	II	S	HL	SxHL
C14:0	0.43 ± 0.03	0.47 ± 0.04	0.38 ± 0.03	0.52 ± 0.04	0.382	0.008	ns
C15:0	0.08 ± 0.01	0.06 ± 0.01	0.06 ± 0.01	0.08 ± 0.01	0.070	0.394	ns
C16:0	19.38 ± 0.49	19.41 ± 0.58	18.52 ± 0.45	20.28 ± 0.62	0.975	0.032	*
C17:0	0.18 ± 0.02	0.16 ± 0.02	0.17 ± 0.02	0.16 ± 0.02	0.563	0.793	ns
C18:0	12.10 ± 0.37	11.23 ± 0.43	10.59 ± 0.34	12.73 ± 0.47	0.135	0.001	*
C20:0	0.13 ± 0.01	0.13 ± 0.01	0.13 ± 0.01	0.13 ± 0.01	1.000	0.849	ns
C22:0	0.04 ± 0.00	0.05 ± 0.00	0.05 ± 0.00	0.05 ± 0.01	0.580	0.595	ns
C14:1	0.03 ± 0.00	0.04 ± 0.00	0.03 ± 0.00	0.04 ± 0.00	0.248	0.354	ns
C16:1	2.29 ± 0.18	2.53 ± 0.22	2.41 ± 0.17	2.40 ± 0.23	0.398	0.997	ns
C20:1	0.26 ± 0.01	0.25 ± 0.02	0.27 ± 0.01	0.25 ± 0.02	0.794	0.434	ns
C22:1	0.05 ± 0.01	0.04 ± 0.01	0.05 ± 0.01	0.04 ± 0.01	0.259	0.115	ns
C18:1n-9	33.77 ± 1.49	34.91 ± 1.76	34.49 ± 1.39	34.20 ± 1.89	0.621	0.904	ns
C18:3n-3	0.95 ± 0.13	1.27 ± 0.16	1.31 ± 0.12	0.92 ± 0.17	0.122	0.074	ns
C20:3n-3	0.02 ± 0.01	0.03 ± 0.01	0.03 ± 0.00	0.03 ± 0.01	0.399	0.805	ns
C20:5n-3	0.62 ± 0.07	0.55 ± 0.08	0.37 ± 0.07	0.81 ± 0.09	0.520	0.001	*
C22:6n-3	1.69 ± 0.17	1.09 ± 0.19	0.98 ± 0.16	1.79 ± 0.21	0.027	0.006	*
C18:2n-6	17.55 ± 1.61	18.34 ± 1.90	20.96 ± 1.50	14.94 ± 2.04	0.749	0.027	ns
C20:3n-6	0.26 ± 0.02	0.24 ± 0.02	0.23 ± 0.02	0.27 ± 0.03	0.677	0.276	ns
C20:4n-6	9.97 ± 0.51	9.02 ± 0.59	8.79 ± 0.47	10.20 ± 0.64	0.229	0.041	ns
SFA	32.35 ± 0.50	31.51 ± 0.58	29.91 ± 0.46	33.96 ± 0.63	0.276	0.0001	*
MUFA	36.41 ± 1.64	37.77 ± 1.93	37.25 ± 1.52	36.93 ± 2.07	0.589	0.904	ns
PUFA	31.24 ± 1.90	30.72 ± 2.24	32.84 ± 1.77	29.1.1 ± 2.40	0.858	0.224	ns
PUFA n-6	27.93 ± 1.84	27.77 ± 2.17	30.14 ± 1.71	25.57 ± 2.33	0.957	0.129	ns
PUFA n-3	3.29 ± 0.27	2.94 ± 0.32	2.69 ± 0.25	3.55 ± 0.35	0.406	0.054	ns
UFA	68.49 ± 0.58	67.65 ± 0.50	70.09 ± 0.46	66.04 ± 0.63	0.277	0.0001	*
Qualitative lipid indices							
PUFA/SFA	0.99 ± 0.07	0.98 ± 0.09	1.11 ± 0.07	0.86 ± 0.10	0.961	0.048	ns
n-6/n-3	9.67 ± 1.30	11.00 ± 1.53	12.60 ± 1.21	8.07 ± 1.65	0.031	0.037	ns
Nutritional lipid indices							
AI	0.31 ± 0.01	0.30 ± 0.01	0.28 ± 0.01	0.34 ± 0.01	0.887	0.003	*
TI	0.76 ± 0.02	0.75 ± 0.03	0.71 ± 0.02	0.81 ± 0.03	0.729	0.021	*
h/H	3.31 ± 0.14	3.30 ± 0.11	3.57 ± 0.11	3.06 ± 0.15	0.980	0.009	*
PI	78.27 ± 3.74	70.65 ± 4.40	70.46 ± 3.47	78.46 ± 4.73	0.018	0.019	ns
NVI	2.39 ± 0.07	2.37 ± 0.06	2.43 ± 0.06	2.32 ± 0.08	0.813	0.282	ns
HPI	3.25 ± 0.13	3.27 ± 0.11	3.53 ± 0.11	2.98 ± 0.14	0.928	0.006	*

* *p* < 0.05; ns = not significant; SxHL = sex and hunting location interaction; SFA = saturated fatty acids; MUFA = monounsaturated fatty acids; PUFA = polyunsaturated fatty acids; UFA = unsaturated fatty acids; PUFA/SFA = ratio of PUFA and SFA; PUFAn-6 = sum of n-6 PUFA; PUFAn-3 = sum of n-3; UFA = unsaturated fatty acids; n-6/n-3 = ratio of n-6 and n-3 fatty acids; AI = atherogenicity index; TI = thrombogenicity index; h/H = hypocholesterolemic/hypercholesterolemic ratio; PI = peroxidability index; NVI = nutritional value index; HPI = health-promoting index.

## Data Availability

The original contributions presented in the study are included in the article, further inquiries can be directed to the corresponding author.
